# School of Science v. Society of Science, Letters, and Art of London

**Published:** 1890-04-05

**Authors:** C. Johnson

**Affiliations:** 4, Ludgate Circus Buildings, London, E.C.


### Editor's Letter-Box.

[Our Correspondents are reminded that prolixity is a great bar to publication, and that brevity of style and conciseness of statement greatly facilitate early insertion.]

### SCHOOL OF SCIENCE v. SOCIETY OF SCIENCE, LETTERS, AND ART OF LONDON.

Referring to your issues of March 15th and 22nd (pp. 375 and viii.), allow me to inform you that the School of Science referred to in March 15th is not in any way connected with the Society of Science, Letters, and Art of London, 160, Holland Road, Kensington. That Dr. C. Waite, who calls himself the late Principal, Trinity College, Wallington, and St. John's College, Penge, is not an Examiner in Roman Law and Equity and Lecturer on Jurisprudence to the Society, and that his name is not to be found in the List of Fellows in the Transactions of the Society from September, 1888, to the present time. That the Society does not examine in Roman Law, Equity, and Jurisprudence.

C. Johnson.

4, Ludgate Circus Buildings, London, E.C.

